# Determinants of burnout in the service of gynecology Mahdia: about 122 cases

**DOI:** 10.1192/j.eurpsy.2022.1520

**Published:** 2022-09-01

**Authors:** N. Faouel, R. Ben Soussia, F. Haboub, M. Kacem, W. Bouali, A. Haj Mohamed, L. Zarrouk

**Affiliations:** 1 hospital Tahar sfar Mahdia, Epartment Of Psychiatry Mahdia, Mahdia, Tunisia; 2 hospital Tahar sfar Mahdia, Epartment Of Psychiatry Mahdia, mahdia, Tunisia; 3 hospital Tahar sfar Mahdia, Epartment Of Psychiatry Mahdia, monastir, Tunisia; 4 hospital Tahar sfar Mahdia, Department Of Psychiatry Mahdia, monastir, Tunisia

**Keywords:** Burnout /Mahdia /Gynecology service

## Abstract

**Introduction:**

The Burnout syndrome occurs preferentially in individuals subjected to intense stress conditions. The nursing staff in Obstetrics Gynecology is an example of this.

**Objectives:**

To assess the prevalence of Burnout and its determinants in the obstetrics gynecology service - Mahdia.

**Methods:**

We conducted an analytical cross-sectional study carried out with the medical and paramedical staff of the gynecology-obstetrics department of the Taher Sfar Mahdia hospital during a period of 3 months.We used a pre-established self-questionnaire containing 2 parts: a part exploring the socio-demographic data of the population and a psychometric part evaluating Burnt out using the “Maslach Burnout” scale inventory ”.

**Results:**

Our sample consisted of 122 medical and paramedical personnel.The sex ratio was 4.3 (99/23), the mean age was 30.5 with values ranging from 25 to 55 years.Of the participants, 59 (48.3%) were single .Nine (7.4%) of the participants were smokers and 2 (1.6%) consumed alcohol.The majority of the population (96.7%) did not have a psychiatric history, 88 (80.3%) reported an organ history.A high level of burnout was noted in 64.8% of our population with 14.8% severe burnout. The presence of burnout was significantly associated with the consumption of psychoactive substances (p = 0.05) and professional rank (p = 0.04) .Nurses, residents and senior doctors werethose most at risk of developing burnout. It was also significantly related to the absence of other professional activities such as research (p = 0.05) and training continuing medical care (p = 0.05).

**Conclusions:**

Psychological intervention strategies with these suffering health promoters would be desirable.

**Disclosure:**

No significant relationships.

